# Establishment of a Novel Risk Score System of Immune Genes Associated With Prognosis in Esophageal Carcinoma

**DOI:** 10.3389/fonc.2021.625271

**Published:** 2021-03-30

**Authors:** Zhenghua Fei, Rongrong Xie, Zhi Chen, Junhui Xie, Yuyang Gu, Yue Zhou, Tongpeng Xu

**Affiliations:** ^1^ Department of Oncology, The First Affiliated Hospital of Wenzhou Medical University, Wenzhou, China; ^2^ Department of Head and Neck Surgery, Tumor Hospital of Ganzhou, Ganzhou, China; ^3^ Department of Thoracic Surgery, The First Affiliated Hospital of Nanjing Medical University, Nanjing, China; ^4^ Department of Oncology, The First Affiliated Hospital of Nanjing Medical University, Nanjing, China

**Keywords:** esophageal carcinoma, histological subtype, immune gene, prognostic model index, risk score, bioinformatics, immunotherapy

## Abstract

**Background:**

Few studies have addressed the role of immune-related genes in the survival and prognosis of different esophageal cancer (EC) sub-types. We established two new prognostic model indexes by bioinformatics analysis to select patients with esophageal squamous cell carcinoma (ESCC) and esophageal adenocarcinoma (EAC) who may benefit from immunotherapy.

**Methods:**

Based on TCGA and ImmPort data sets, we screened immune genes differentially expressed between tumor and normal tissues in ESCC and EAC and analyzed the relationship between these genes and patient survival outcomes. We established the risk score models of immune-related genes in ESCC and EAC by multivariate COX regression analysis.

**Results:**

We identified 12 and 11 immune-related differentially expressed genes associated with the clinical prognosis of ESCC and EAC respectively, based on which two prognostic risk score models of the two EC sub-types were constructed. It was found that the survival probability of patients with high scores was significantly lower than that of patients with low scores (p < 0.001). BMP1, EGFR, S100A12, HLA-B, TNFSF18, IL1B, MAPT and OXTR were significantly related to sex, TNM stage or survival outcomes of ESCC or EAC patients (p < 0.05). In addition, the risk score of ESCC was significantly correlated with the level of B cell infiltration in immune cells (p < 0.05).

**Conclusions:**

The prognosis-related immune gene model indexes described herein prove to be useful prognostic biomarkers of the two EC sub-types in that they may provide a reference direction for looking for the beneficiaries of immunotherapy for EC patients.

## Introduction

Esophageal cancer (EC) is the seventh most common cancer and the sixth leading cause of cancer death worldwide, with a 5-year survival rate of lower than 20%, seriously affecting human health (1, 2). In 2017, the number of global new EC cases and deaths is 473000 and 436000, respectively (3). It is estimated that there will be about 18440 diagnosed EC cases in 2020 causing about 16170 deaths in the United States (4). The treatment of EC mainly focuses on preoperative chemotherapy or postoperative radiotherapy and chemotherapy. However, about 50% EC patients respond unsatisfactorily to the treatment due to resistance of cancer cells to the chemotherapeutic drugs (5). Esophageal squamous cell carcinoma(ESCC) and esophageal adenocarcinoma (EAC) are two pathological types of EC characterized by different distribution, etiology and risk factors (6, 7). In addition, they vary in molecular characteristics and undergo different changes in their specific genes (8).

In recent years, immunotherapy has emerged as a new treatment for various malignant tumors including EC, knowing that it plays a role in immunosuppression in the tumor micro-environment. Several clinical trials have shown that immune checkpoint inhibitors (ICI) nivolumab and Pembrolizumab can be regarded as new standard second-line treatment strategies for EC (9–14). ICIs include anti-PD1, anti-PD-L1, and anti-CTLA-4 (15). However, only a small number of patients can clinically benefit from ICI treatment because of drug resistance, and some patients even deteriorate sharply after immunotherapy. Therefore, it is necessary to identify biomarkers that can accurately predict patients who could benefit from immunotherapy for the sake of providing individualized treatment (9).

At present, microsatellite instability (MSI), PD-L1, tumor mutation burden (TMB), DNA mismatch repair deficiency (dMMR) and TILs factors are the only factors that are confirmed to be able to predict the efficacy of ICI response (16, 17). One study identified that immune-related genes (ABL1, ATF2, ATG5, C6, CD38, HMGB1, ICOSLG, IL12RB2, and PLAU) were significantly associated with overall survival (OS) of ESCC patients (18). With the emergence of open data sets of gene expression, some studies established immune gene prognostic markers for predicting the survival of EC patients (19). A large-scale multicenter retrospective study analyzed and established immune markers based on four genes (SERPINE1, MMP12, PLAUR and Eps8) in predicting pathological complete remission of neoadjuvant radiotherapy and chemotherapy in EC patients, which is believed to lay a foundation for the combination of neoadjuvant radiotherapy and chemotherapy with immunotherapy (20). The study of Lu et al. showed that CD103+CD8+TIL displayed the tissue-resident memory T cell phenotype and showed high expression of immune checkpoint (PD-1, TIM-3) in ESCC. After blocking anti-PD-1, CD103+CD8+TIL induced strong anti-tumor immunity (21). To help find individual immunotherapy targets, some recent studies established comprehensive prognostic indicators of lung squamous cell carcinoma, colorectal cancer and other tumors based on immune genome map analysis (22–29). However, the prognostic value of immune-related genes in ESCC and EAC as two pathological sub-types of EC has not been fully elucidated.

Using TCGA and ImmPort database, we combined the patient’s clinical information with an immune-related genomic map, and found immune-related genes that were significantly related to prognosis with ESCC and EAC respectively. Based on these genes, we constructed individual immune prognostic index models for EC patients, hoping that they could lay a foundation for promoting individualized immunotherapy of EC.

## Materials and Methods

### Data Collection and Processing

**Figure 1:**
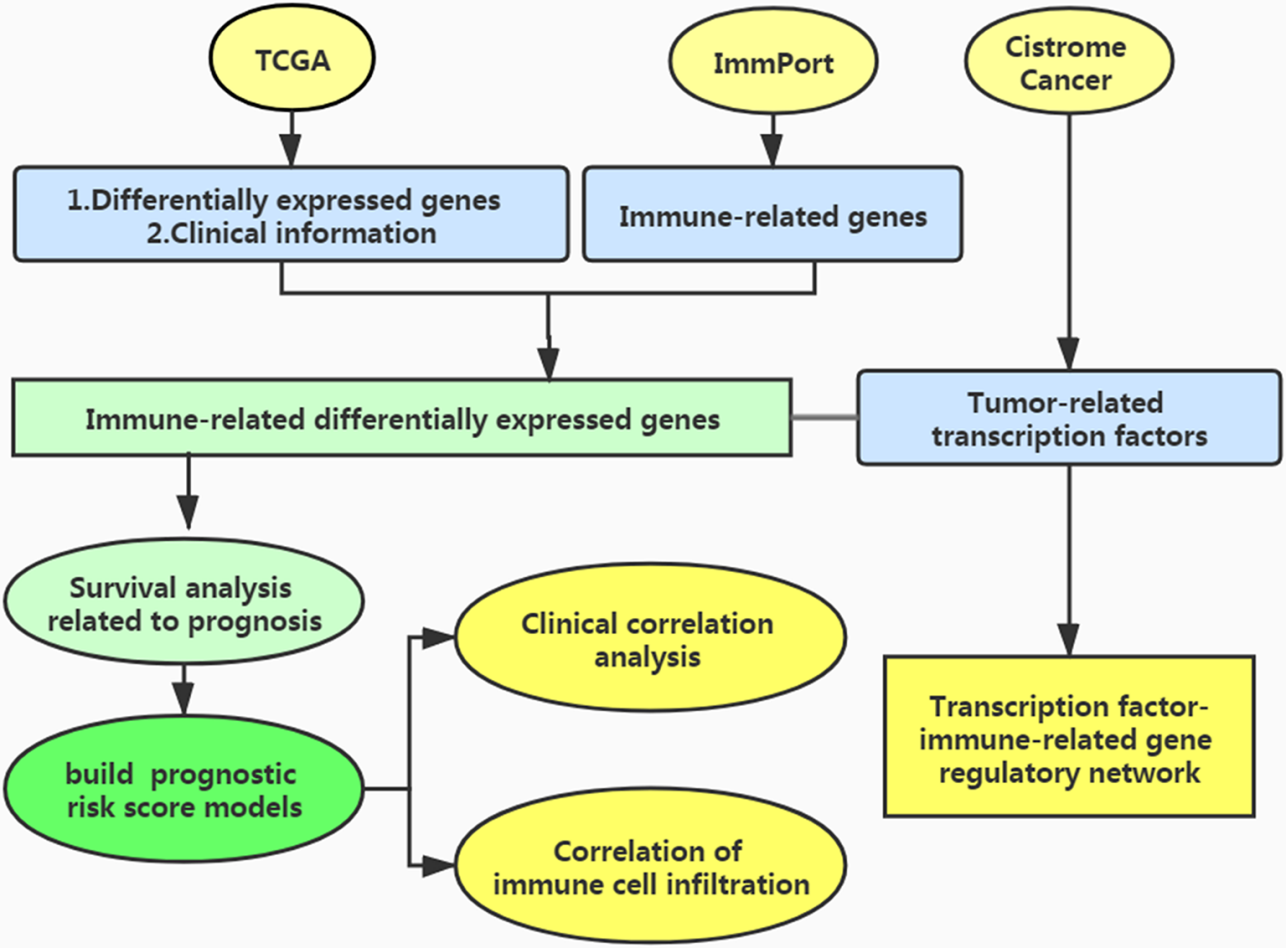
Work flow of the study.

The process of this study is shown in **Figure 1**. We used the TCGA database (https://cancergenome.nih.gov/) to obtain immune-related gene transcriptome data, clinical data, and follow-up data of EC patients. In the ImmPort data portal (https://www.immport.org/), the list of immune-related genes was derived.

### Screening of Differentially Expressed Genes

We used the edgeR package to find differentially expressed genes, using |logFC|>1 and FDR < 0.05 as the screening criteria.

### Identification of Differentially Expressed Immune-Related genes

We crossed the above differentially expressed genes with immune genes and used the edgeR software package of R software (http://bioconductor.org/Packages/edger/) and Wilcoxon test analysis to obtain the immune differentially expressed genes related to EC.

### TF Analysis and Construction of the Regulatory Network of TF-Immune-Related Genes

To explore the regulatory mechanism of immune-related genes related to survival, we searched the Cistrome Cancer database (http://cistrome.org), downloaded tumor-related transcription factors(TFs), and extracted differentially expressed TFs related to clinical prognosis. We constructed an interaction network between these TFs and immune differential genes related to prognosis to explore the mechanism of TF in regulating these genes.

### Analysis of Survival-Associated Immune-Related Genes

The patients were randomly divided into two groups: training group and testing group. Using the R software survival software package, we carried out univariate Cox analysis in training group to explore the immune-related genes related to survival of EC patients by integrating the differential immunity-related genes with the survival data of the EC patients.

### Development of the Immune Gene Prognosis Model in Training Group

Using multivariate Cox regression, we established an evaluation model of prognostic risk indexes based on EC immune-related genes and calculated the risk indexes using the following equation in training group: Risk score= coefficient of multivariate Cox regression(a) × gene expression level(a) + coefficient of multivariate Cox regression(b) × gene expression level(b) + … + coefficient of multivariate Cox regression(n) × gene expression level(n).

### Analysis of the Survival Differences Between High- and Low-Risk Patients

Based on the median score of differential immune gene risk scores related to survival, the patients in training group were divided into high- and low-risk groups. Survival curves were mapped out to explore differences in survival prognosis between the two groups. The survival ROC R software package was used to draw the receiver operating characteristic (ROC) and calculated the area under the AUC curve of the patients to judge the true positive rate. Using the Survminer software package of R software, survival prognosis of the patients in the two groups was assessed.

### Validation of the Immune Gene Prognosis Model in Testing Group and Entire Group

We verified the reliability of the prognostic score formula in the testing group and the entire group. We used the formula in the training group to calculate the risk score of each patient in the testing group and the entire group, and divided them into high-risk group and low-risk group respectively. Similarly, the ROC curves of the testing group and the entire group were drawn to evaluate the prognostic value of the model.

### Clinical Relevance of the Clinical Characteristics

Using the calculated risk score, the clinical correlation between survival-related immune genes and the patient clinical data including sex and tumor stage was evaluated. Using the ggpubr software package, the clinical correlation between the survival-related immune genes and the clinical data including gender and tumor stage was explored.

### Evaluation of Immune Cell Infiltration and the Tumor Microenvironment

The data of immune cell infiltration in EC patients were extracted from the Timer database (https://cistrome.shinyapps.io/timer/) to explore whether the risk score was related. The immune cells included B cells, CD4+T cells, CD8+T cells, neutrophils, dendritic cells, and macrophages.

### Statistical Analysis

Differences in immune genes were analyzed using R software (version 3.6.1). The expression of immune-related genes and their relationships with survival were analyzed using one-way ANOVA. The classified variables were determined by the χ 2 test, and the variables with p < 0.05 were subjected to multivariate analysis. Univariate and multivariate Cox regression analyses were used to explain clinicopathological features and risk scores on prognosis. The survival ROC R package was used to calculate the ROC curve and evaluate the accuracy of the immune-related gene prediction index. Differences in clinical parameters were analyzed by independent t-test. p < 0.05 was considered statistically significant. The Kaplan-Meier curve was established by R software survival and Survminer software package. Bilateral Log Rank test and Kaplan-Meier method were used to explore the survival of EC patients in high- and low-risk groups.

## Results

### Identification of Differentially Expressed Immune-Related Genes

**Figure 2:**
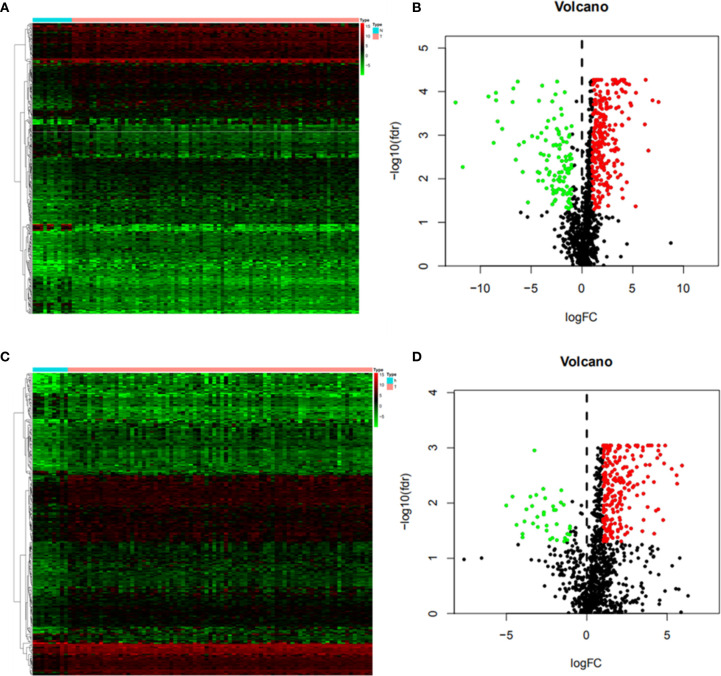
Differential immune genes **(A)** Heat map of esophageal squamous cell carcinoma(ESCC); **(B)** Volcanic map of ESCC; **(C)** Heat map of esophageal adenocarcinoma (EAC); **(D)** Volcanic map of EAC. The Abscissa of the heat map shows the normal tissue (blue) and esophageal cancer tissue (red), and the ordinate shows the genes. In the volcano map, the blue green and black colors indicate upregulated genes, down-regulated genes and genes with no significant difference respectively.

Differentially expressed genes were screened between EC and normal tissues from the TCGA database. It was found that 5942 genes were differentially expressed in ESCC, including 4283 up-regulated and 1659 down-regulated genes. Using the same method, 3026 upregulated genes and 625 down-regulated genes were screened in EAC. By crossing these genes with immune genes, we obtained 372 immune-related differential genes in ESCC, of which 257 were up-regulated and 115 were down-regulated (**Figures 2A, B**). In EAC, 232 immune-related differential genes were upregulated and 37 were down-regulated (**Figures 2C, D**).

### TF Analysis and Construction of the TF-Immune-Related Gene Regulatory Network

**Figure 3:**
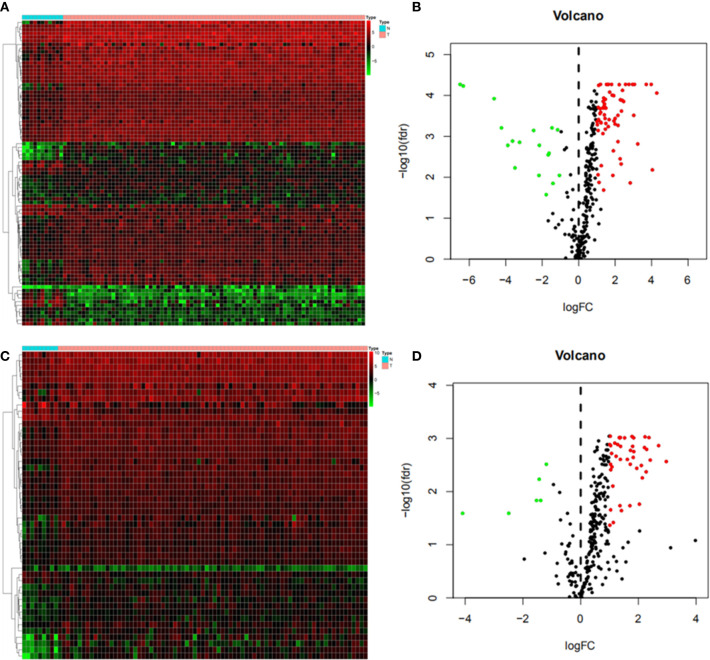
Differential gene expression of immune-related transcription factors **(A)** Heat map of esophageal squamous cell carcinoma (ESCC); **(B)** Volcanic map of ESCC; **(C)** Heat map of esophageal adenocarcinoma (EAC); **(D)** Volcanic map of EAC.

We downloaded 83 and 49 different TFs between the two EC subtypes and normal tissues in the Cistrome database, and found that 65 were upregulated and 18 were down-regulated in ESCC (**Figures 3A, B**), and 43 were upregulated and 6 were down-regulated in EAC (**Figures 3C, D**) respectively.

**Figure 4:**
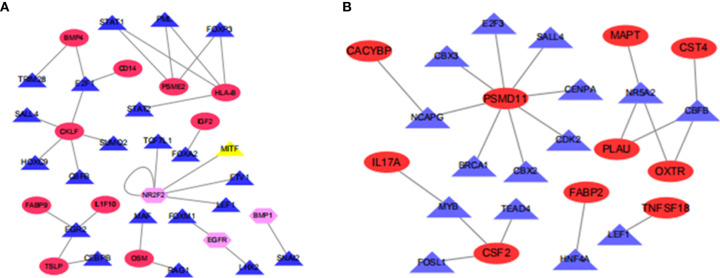
Map of transcriptional factors regulating the immune gene network. **(A)** Esophageal squamous cell carcinoma; **(B)** Esophageal adenocarcinoma.

To understand the regulatory mechanism of these immune differential genes related to prognosis, we developed a regulatory network of TF-immune gene interaction based on these differential TFs and our previously screened immune differential genes, finding that BMP4, CD14, PSME2, HLA-B, IGF2, CKLF, FABP9, IL1F10, TSLP, and OSM were high-risk immune genes while NR2F2, EGFR, and BMP1 were low-risk immune genes in ESCC (**Figure 4A**). CACYBP, MAPT, CST4, PSMD11, IL17A, PLAU, OXTR, FABP2, CSF2, and TNFSF18 were high-risk immune genes in EAC (**Figure 4B**).

### Analysis of Survival-Associated Immune-Related Genes

**Figure 5:**
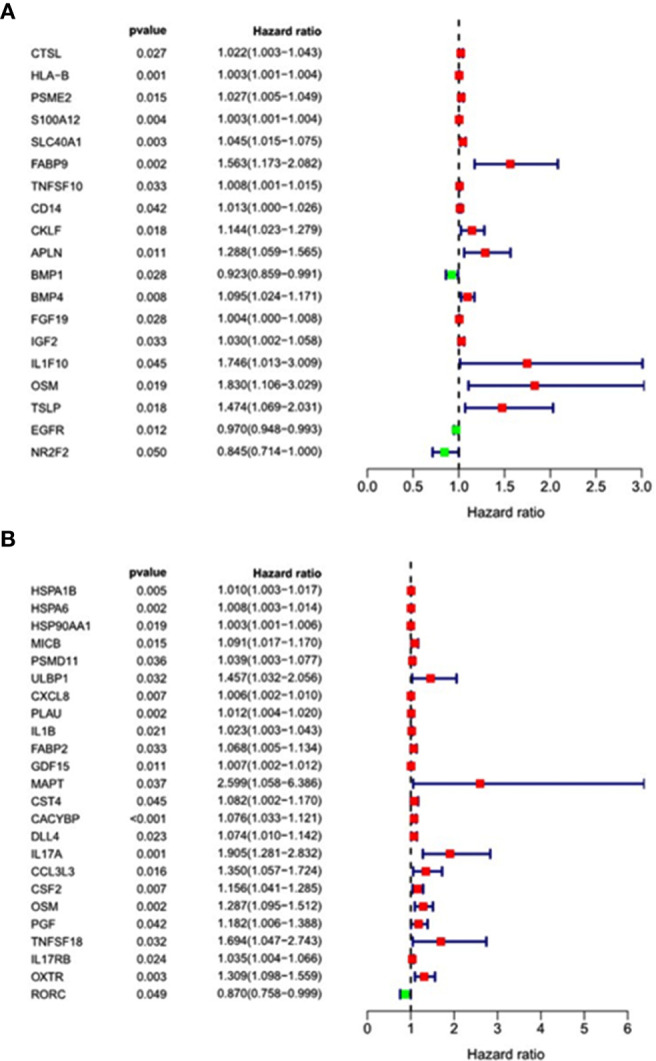
Forest map of univariate analysis of prognosis-related immune genes. **(A)** Esophageal squamous cell carcinoma; **(B)** Esophageal adenocarcinoma.

ESCC and EAC patients with complete clinical data were randomly divided into training groups and testing groups. In training groups, we combined the above screened immune-related differentially expressed genes with the clinical information and follow-up data of the patients for survival analysis. To obtain the immune-related differential genes related to prognosis, we used univariate analysis of prognosis-related immune genes(p < 0.05). The results revealed that the 16 genes (CTSL, S100A12, SLC40A1, BMP4, FGF19, TNFSF10, CD14, PSME2, HLA-B, APLN, IGF2, CKLF, FABP9, IL1F10, TSLP, and OSM) were high-risk genes, while NR2F2, EGFR and BMP1 were low-risk genes in ESCC (**Figure 5A**). Besides, there were 23 high-risk genes and 1 low-risk gene in EAC (**Figure 5B**).

### Development of the Immune Gene Prognosis Model in Training Group

Based on the immune differential genes related to prognosis and the survival data of patients, we used multivariate COX regression analysis to construct a prognosis model of immune-related genes. Univariate and multivariate COX regression analyses showed that 12 and 11 genes were included in our prognostic model for ESCC and EAC, respectively. The formula for calculating the risk score is as follows: Risk score of ESCC = 0.006486756 × expression level of HLA-B + 0.003511031 × expression level of S100A12 - 0.134223077 × expression level of SLC40A1 + 0.785407491 × expression level of FABP9 - 0.021149907 × expression level of CD14 + 0.238292334 × expression level of APLN - 0.226841694 × expression level of BMP1 + 0.315827065 × expression level of BMP4 + 0.00809996 × expression level of FGF19 + 0.118629975 × expression level of IGF2 + 1.008924188 × expression level of OSM - 0.01772927 × expression level of EGFR (**Table 1A**). Risk score of EAC = - 1.230594393 × expression level of ULBP1 + 0.060399108 × expression level of IL1B + 0.163713621 × expression level of FABP2 + 2.31747042 × expression level of MAPT + 0.139658347 × expression level of CST4 + 0.132029819 × expression level of CACYBP + 0.156437833 × expression level of DLL4 + 1.690819322 × expression level of IL17A - 0.428632286 × expression level of PGF + 0.943049137 × expression level of TNFSF18 + 0.568394224 × expression level of OXTR (**Table 1B**).

**Table 1A:** The coefficients and HR values of the immune gene prognostic model of esophageal squamous cell carcinoma.

ID	Coefficient	HR	HR.95L	HR.95H	P-value
**HLA-B**	0.006486756	1.006507841	1.003310694	1.009715175	6.44E-05
**S100A12**	0.003511031	1.003517202	1.000985994	1.006054811	0.00643444
**SLC40A1**	-0.134223077	0.874394986	0.787697531	0.970634743	0.011754987
**FABP9**	0.785407491	2.193300509	1.428686843	3.367124957	0.00032917
**CD14**	-0.021149907	0.979072184	0.956722516	1.001943955	0.07263327
**APLN**	0.238292334	1.269080134	0.963370653	1.671801378	0.090153576
**BMP1**	-0.226841694	0.79704695	0.687860206	0.923565334	0.00254644
**BMP4**	0.315827065	1.371393073	1.126086446	1.670137286	0.00168415
**FGF19**	0.00809996	1.008132853	1.002605222	1.01369096	0.003883631
**IGF2**	0.118629975	1.125953211	1.061553465	1.194259804	7.89E-05
**OSM**	1.008924188	2.742648851	1.299469363	5.788611056	0.008113535
**EGFR**	-0.01772927	0.982426969	0.957043498	1.00848368	0.18436175

**Table 1B:** The coefficients and HR values of the immune gene prognostic model of esophageal adenocarcinoma.

ID	Coefficient	HR	HR.95L	HR.95H	P-value
**ULBP1**	-1.230594393	0.292118893	0.126723565	0.673382631	0.003877009
**IL1B**	0.060399108	1.062260419	1.034611003	1.09064875	7.17E-06
**FABP2**	0.163713621	1.177876947	1.094446903	1.267666891	1.26E-05
**MAPT**	2.31747042	10.14996665	2.498644078	41.23109167	0.001193568
**CST4**	0.139658347	1.149880871	1.015526482	1.302010377	0.027594744
**CACYBP**	0.132029819	1.141142347	1.074438638	1.211987182	1.74E-05
**DLL4**	0.156437833	1.169338066	1.029335181	1.328383153	0.016201768
**IL17A**	1.690819322	5.423922827	2.745975149	10.71347599	1.12E-06
**PGF**	-0.428632286	0.651399414	0.447948699	0.947253998	0.024858035
**TNFSF18**	0.943049137	2.567799064	1.368401511	4.818462988	0.003317824
**OXTR**	0.568394224	1.76542989	1.300097375	2.397314814	0.000271409

### Analysis of Survival Differences Between High- and Low-Risk Patients

**Figure 6:**
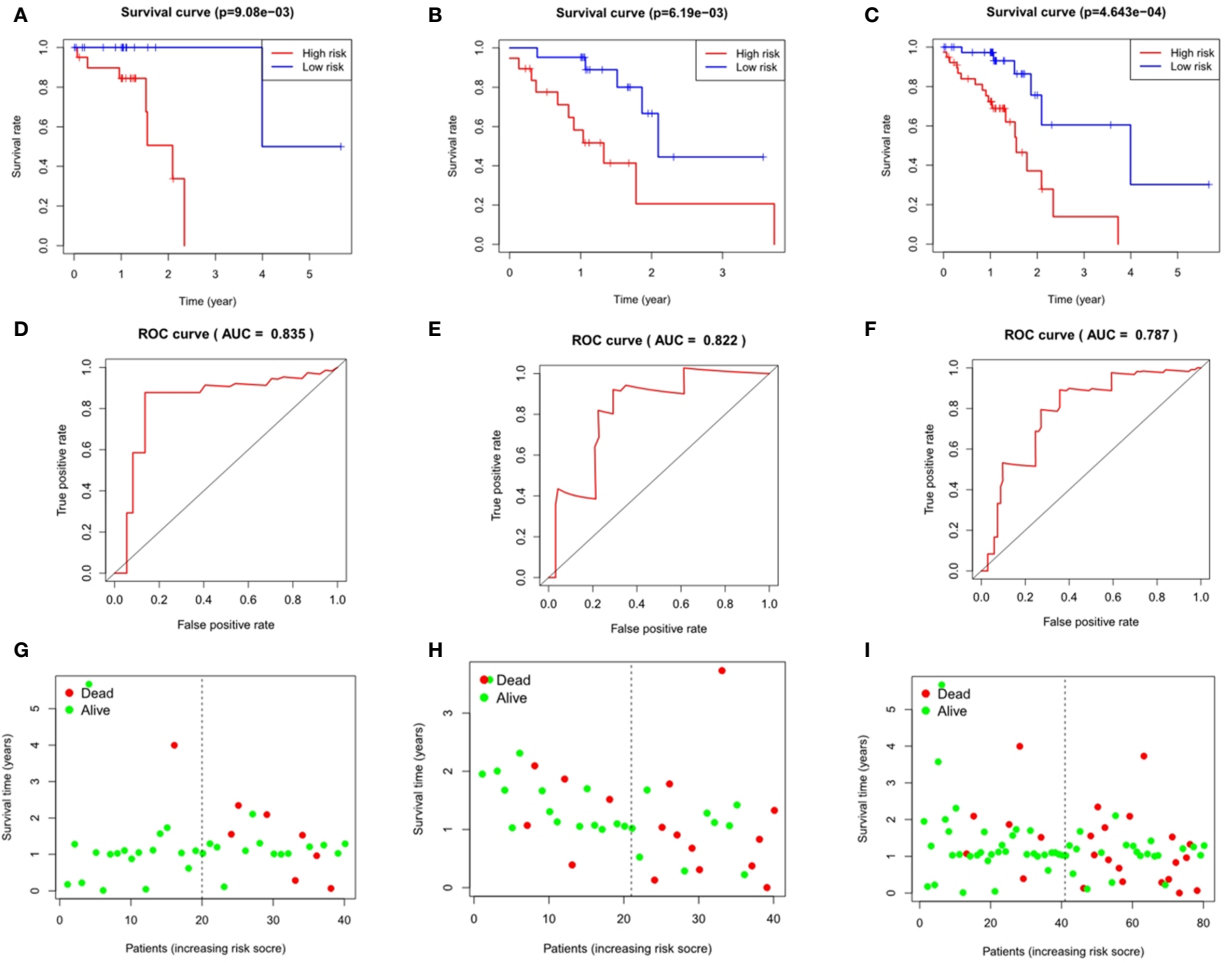
Evaluation and verification of the model in esophageal squamous cell carcinoma. Kaplan-Meier analysis in high and low risk groups in the train group **(A)**, testing group **(B)** and entire group **(C)**; ROC curve in the train group **(D)**, testing group **(E)** and entire group **(F)**; survival status in the train group **(G)**, testing group **(H)** and entire group **(I)**.

**Figure 7:**
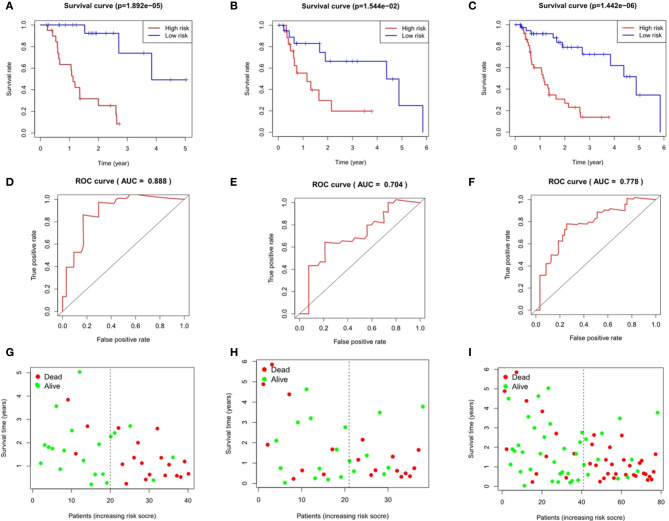
Evaluation and verification of the model in esophageal adenocarcinoma. Kaplan-Meier analysis in high and low risk groups in the train group **(A)**, testing group **(B)** and entire group **(C)**; ROC curve in the train group **(D)**, testing group **(E)** and entire group **(F)**; survival status in the train group **(G)**, testing group **(H)**, and entire group **(I)**.

Taking the median as the demarcation, we divided the patients into a high-risk group and a low-risk group according to the risk score, and found that survival prognosis of the patients in the high-risk group was significantly worse than that in the low-risk group(p < 0.001)(**Figures 6A** and **7A**). Under the ROC curves, the area was 0.835 in ESCC and 0.888 in EAC, showing high true positive rates(**Figures 6D** and **7D**). The number of deaths in the high-risk group was significantly higher than that in the low-risk group, and the survival time was also significantly shorter than that in the low-risk group (**Figures 6G** and **7G**).

**Figure 8:**
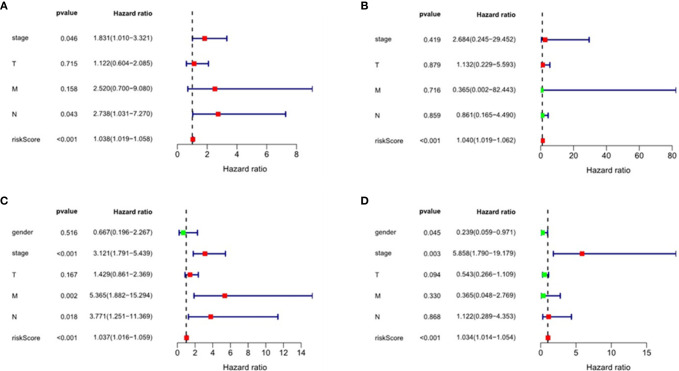
Univariate and multivariate regression analyses of esophageal squamous cell carcinoma(ESCC) and esophageal adenocarcinoma (EAC). Univariate regression analysis: **(A)** ESCC; **(C)** EAC. Multivariate regression analysis: **(B)** ESCC; **(D)** EAC.

We performed multivariate Cox regression analysis to adjust gender, tumor stage, tumor size, lymph node metastasis status, distant metastasis, and other factors (**Figure 8** and **Table 2**). The analysis chart shows that the risk score could independently predict the prognosis of both ESCC and EAC patients (p < 0.001).

Table 2AUnivariate and multivariate regression analysis of independent prognostic factors of esophageal squamous cell carcinoma.

**Table:** 

Variables	Univariate analysis			Multivariate analysis		
	HR	95% CI	P value	HR	95% CI	P value
**Stage**	1.831	(1.010-3.321)	0.046	2.684	(0.245-29.452)	0.419
**T**	1.122	(0.604-2.085)	0.715	1.132	(0.229-5.593)	0.879
**M**	2.520	(0.700-9.080)	0.158	0.365	(0.002-82.443)	0.716
**N**	2.738	(1.031-7.270)	0.043	0.861	(0.165-4.490)	0.859
**Riskscore**	1.038	(1.019-1.058)	<0.001	1.040	(1.019-1.062)	<0.001

**Table 2B:** Univariate and multivariate regression analysis of independent prognostic factors of esophageal adenocarcinoma.

Variables	Univariate analysis			Multivariate analysis		
	HR	95% CI	P value	HR	95% CI	P value
**Gender**	0.667	(0.196-2.267)	0.516	0.239	(0.059-0.971)	0.045
**Stage**	3.121	(1.791-5.439)	<0.001	5.858	(1.790-19.179)	0.003
**T**	1.429	(0.861-2.369)	0.167	0.543	(0.266-1.109)	0.094
**M**	5.365	(1.882-15.294)	0.002	0.365	(0.048-2.769)	0.330
**N**	3.771	(1.251-11.369)	0.018	1.122	(0.289-4.353)	0.868
**Riskscore**	1.037	(1.016-1.059)	<0.001	1.034	(1.014-1.054)	<0.001

### Validation of the Immune Gene Prognosis Model in Testing Group and Entire Group

The area under the ROC curves of the testing group and the entire group of ESCC was 0.822 and 0.787 respectively (**Figures 6E, F**), and that of EAC was 0.704 and 0.778 respectively (**Figures 7E, F**), which showed good predictive ability. The Kaplan-Meier curve showed us the difference in survival rate between the high-risk group and the low-risk group. For ESCC, the results showed that the overall survival rate of the high-risk group was significantly lower than that of the low-risk group in both the testing group (p = 6.19e-03, **Figure 6B**) and the entire group (p = 4.643e-04, **Figure 6C**). In the case of EAC, we came to the same conclusion(p = 1.544e-02, **Figure 7B**; p = 1.442e-06, **Figure 7C**). **Figures 6H** and **7H** show the survival states in the testing cohort and **Figures 6I** and **7I** show that in the entire cohort, in ESCC and EAC, respectively.

### Clinical Relevance of the Clinical Characteristics

**Figure 9:**
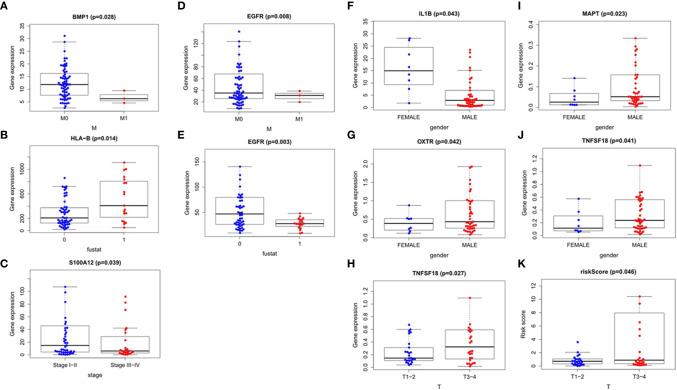
Correlation analysis of the clinical factors. Esophageal squamous cell carcinoma: **(A–E)** Esophageal adenocarcinoma: **(F–K)**.

We also explored the relationship between the risk score, immune-related differential genes and clinical characteristics of the tumors(p < 0.05) (**Table 3**). The results showed that the expression of immune-related differential gene MAPT, TNFSF18, and OXTR in EAC in males were higher than that in females (**Figures 9G, I, J**), and vice versa for the expression of IL1B (**Figure 9F**). The S100A12 gene expression was associated with the tumor stage in ESCC patients (**Figure 9C**). Its expression in stage 3-4 EC was lower than that in stage 1 and 2. In the T stage, the expression of TNFSF18 in the 3-4 stage was significantly higher than that in the 1-2 stage in EAC (**Figure 9H**). However, ESCC patients enriched with gene BMP1 and EGFR were often accompanied with a better M stage, devoid of distant metastasis (**Figures 9A, D** **)**. The study also found a significant positive correlation between our model risk score and the T stage in EAC (**Figure 9K**). In terms of the survival outcome of ESCC, HLA-B was a risk gene, and its high expression indicated a poor survival outcome (**Figure 9B**), while the higher the expression level of EGFR, the greater the likelihood of survival (**Figure 9E**).

Table 3ARelationships between the expression of immune-related genes and the clinicopathological factors in esophageal squamous cell carcinoma.

**Table:** 

ID	Gender (male/female)		Tumor stage (III-IV/I-II)		T (T3–4/T1-2)		M (M1/M0)		N (N1-3/N0)		Fustat (1/0)	
	t	p	t	p	t	p	t	p	t	p	t	p
**HLA-B**	-0.644	(0.530)	-0.798	(0.430)	0.194	(0.847)	-0.576	(0.620)	-0.975	(0.334)	-2.668	**(0.014)**
**S100A12**	0.636	(0.534)	2.122	**(0.039)**	0.254	(0.800)	0.908	(0.385)	1.278	(0.207)	-1.071	(0.297)
SLC40A1	-1.28	(0.215)	-1.55	(0.133)	-1.246	(0.218)	-0.664	(0.573)	-1.577	(0.123)	-1.15	(0.261)
FABP9	-1.604	(0.114)	0.81	(0.422)	1.055	(0.299)	1.433	(0.159)	0.946	(0.350)	-1.054	(0.306)
CD14	-1.924	(0.060)	-1.16	(0.257)	-0.937	(0.353)	1.114	(0.313)	-0.983	(0.332)	-0.929	(0.364)
APLN	-0.849	(0.406)	-0.581	(0.564)	-0.263	(0.793)	0.13	(0.908)	-0.039	(0.969)	-0.919	(0.368)
**BMP1**	-0.008	(0.994)	1.224	(0.226)	-0.161	(0.872)	3.602	**(0.028)**	1.805	(0.076)	1.731	(0.090)
BMP4	-0.068	(0.946)	-1.54	(0.136)	-1.929	(0.060)	1.689	(0.149)	-1.287	(0.207)	-1.229	(0.234)
FGF19	0.501	(0.625)	-0.733	(0.470)	-1.354	(0.184)	1.787	(0.078)	-0.591	(0.558)	-0.844	(0.409)
IGF2	-0.304	(0.765)	-0.738	(0.465)	-0.585	(0.561)	-0.66	(0.577)	0.096	(0.924)	0.599	(0.554)
OSM	-0.318	(0.755)	-0.071	(0.944)	0.319	(0.751)	1.236	(0.323)	0.149	(0.882)	-0.4	(0.693)
**EGFR**	1.079	(0.303)	1.19	(0.238)	-0.264	(0.793)	2.766	**(0.008)**	1.425	(0.160)	3.16	**(0.003)**
Riskscore	-1.657	(0.103)	0.658	(0.513)	-0.59	(0.558)	-0.495	(0.663)	0.855	(0.397)	-1.798	(0.089)

*The value in bold is statistically significant.

**Table 3B:** Relationships between the expression of immune-related genes and the clinicopathological factors in esophageal adenocarcinoma.

ID	Gender (male/female)		Tumor stage (III-IV/I-II)		T (T3–4/T1-2)		M (M1/M0)		N (N1-3/N0)		Fustat (1/0)	
	t	p	t	p	t	p	t	p	t	p	t	p
ULBP1	0.888	(0.403)	-0.264	(0.793)	-0.15	(0.881)	0.577	(0.577)	0.399	(0.694)	-0.222	(0.825)
**IL1B**	2.252	**(0.043)**	-0.928	(0.360)	-0.51	(0.613)	-1.769	(0.150)	0.692	(0.492)	-1.617	(0.114)
FABP2	-0.153	(0.880)	-0.19	(0.850)	0.334	(0.740)	1.304	(0.213)	-0.07	(0.944)	-0.682	(0.499)
**MAPT**	-2.354	**(0.023)**	-0.996	(0.327)	-0.902	(0.373)	2.05	(0.046)	-2.001	(0.052)	0.059	(0.953)
CST4	-1.364	(0.178)	-1.625	(0.116)	-1.719	(0.096)	0.626	(0.542)	-1.901	(0.065)	-1.139	(0.265)
CACYBP	-1.47	(0.151)	-2.139	(0.039)	-1.545	(0.130)	-1.284	(0.263)	-2.292	(0.026)	-2.057	(0.047)
DLL4	-0.058	(0.955)	-1.598	(0.120)	-1.082	(0.285)	0.349	(0.738)	-2.028	(0.048)	-1.833	(0.075)
IL17A	1.063	(0.321)	-0.166	(0.868)	0.156	(0.877)	-0.792	(0.467)	-1.194	(0.238)	-2.042	(0.051)
PGF	0.199	(0.844)	-1.619	(0.116)	-1.64	(0.110)	-0.203	(0.847)	-1.914	(0.061)	-1.69	(0.101)
**TNFSF18**	-2.159	**(0.041)**	-1.472	(0.149)	-2.316	**(0.027)**	-0.368	(0.731)	-0.511	(0.613)	-0.552	(0.584)
**OXTR**	-2.087	**(0.042)**	-2.003	(0.056)	-1.718	(0.096)	0.005	(0.996)	-2.005	(0.052)	-1.589	(0.124)
RiskScore	-1.619	(0.112)	-2.181	(0.039)	-2.091	**(0.046)**	-0.719	(0.508)	-2.254	(0.031)	-2.105	(0.045)

*The value in bold is statistically significant.

### Evaluation of Immune Cell Infiltration and the Tumor Microenvironment

**Figure 10:**
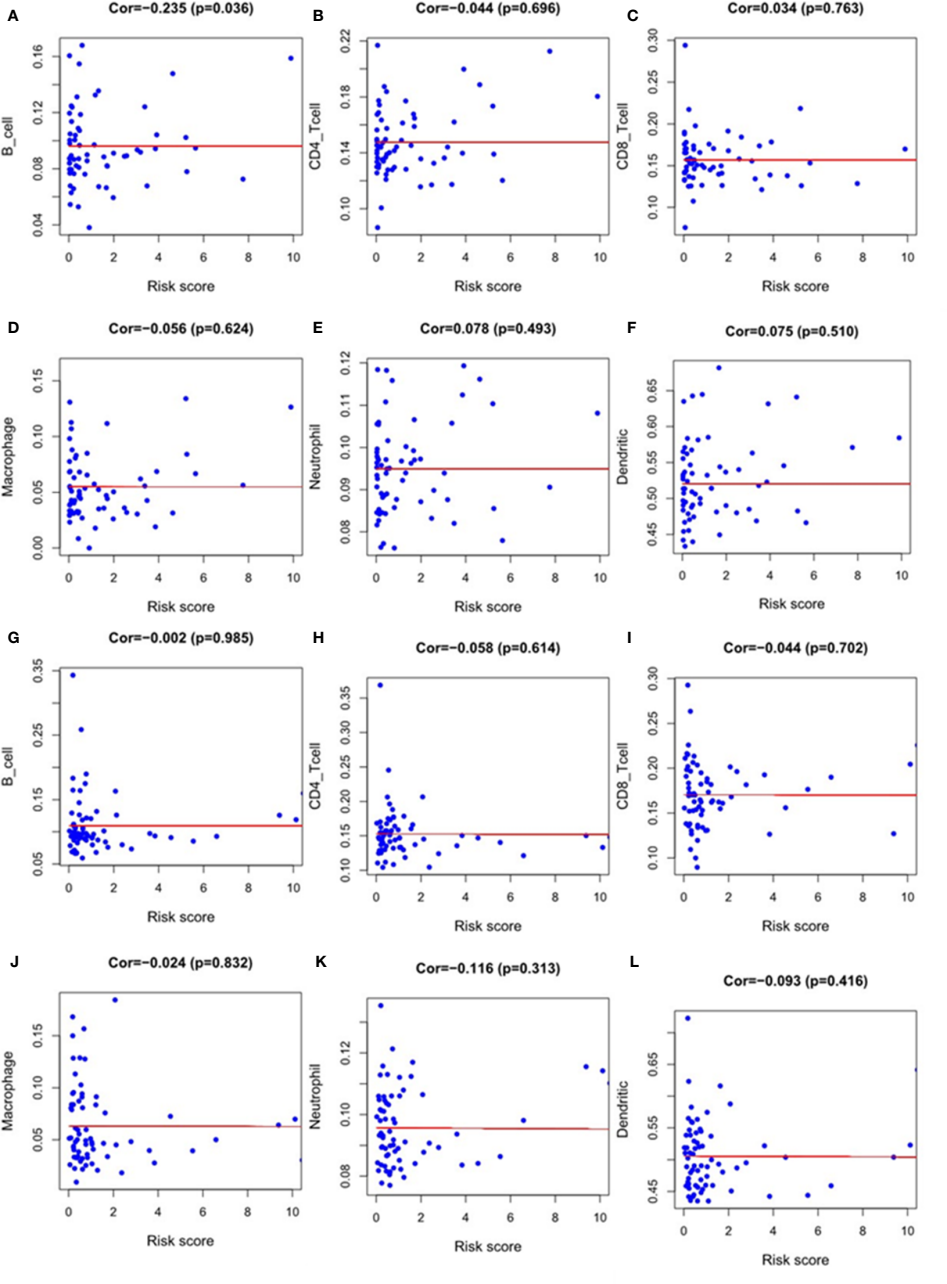
The relationship between the model index and the level of immune cell infiltration, including B-cell, CD4-T cell, CD8-T cell, macrophage, neutrophil and dendritic cell. Esophageal squamous cell carcinoma: **(A–F);** Esophageal adenocarcinoma: **(G–L)**.

We used Timer samples to explore whether the immune genome could indicate the tumor immune microenvironment status in EC patients. We downloaded the immune infiltration level in EC patients, analyzed and visualized the correlation between prognosis-related immune genes and immune cell abundance using TIMER database. The result showed that the level of B cell infiltration was negatively correlated with the score of our immune differential gene evaluation model related to the prognosis of ESCC(p < 0.05)(**Figure 10A**). We found no other statistical correlation between this prognostic index and six kinds of immune cell infiltration(**Figures 10B–L**).

## Discussion

In recent years, immunotherapy has become a research hotspot in cancer therapy. It is necessary to explore the indicators to predict the prognosis of patients on immunotherapy for the sake of screening out immunotherapy beneficiaries. So far, we found no clear consensus biomarker to predict the efficacy of immunotherapy for EC. Few studies have classified the results of immune-related genes in EC according to its histological sub-types. In this study, we analyzed the whole genomes of ESCC and EAC in the TCGA database and established the immune gene prognostic models related to the prognosis of ESCC and EAC based on respective 12 and 11 genes. In addition, we explored the clinical significance of these immune-related genes and their correlations with immune cell infiltration with a view to improve the efficacy of immunotherapy and promote the development of individualized immunotherapy for EC patients.

Genome analysis strongly suggests that ESCC and EAC are different tumor entities, and their genetic change profiles are very different (8, 30). A study on the genomic characteristics of EC found that ESCC and EAC had a set of driving genes that were almost mutually exclusive, indicating that their development was independent (31). Our univariate COX regression analysis showed that 19 and 24 differential immune genes were related to the prognosis of ESCC and EAC respectively. Among them, we mainly analyzed four genes (EGFR and BMP1 in ESCC and IL-1B and MAPT in EAC) that were related to gender, tumor stage, lymph node metastasis, distant metastasis, and other clinical features in EC, supposing that they might play essential roles in the prognosis of EC patients.

The overexpression of epidermal growth factor receptor (EGFR) in ESCC often indicates a poor prognosis (32). It was reported that an ESCC-targeted antibody could improve the radiosensitivity of recurrent ESCC with overexpression of EGFR, suggesting an effective treatment (33). In their *in vitro* and *in vivo* model study, Hoi and his team found that chemotherapy upregulated the expression of PD-L1 in ESCC by activating the EGFR/ERK pathway, suggesting that anti-PD-L1 immunotherapy combined with conventional chemotherapy could achieve a better therapeutic effect (5). Another study showed a correlation between the expression of PD-L1 and EGFR in ESCC, as well as a negative correlation between them on tumor cells or tumor-infiltrating immune cells in ESCC (34). However, few studies focused on the relationship between EGFR gene expression in EC and the prognosis of patients undergoing immunotherapy. Our study found that EGFR was an immune differential gene in patients with ESCC, and its down-regulation was associated with poor prognosis. The expression of EGFR in patients without distant metastasis was higher than that in patients with distant metastasis, and its high expression often indicated a better survival outcome. Our study identified that BMP1 was also an immune differential gene related to prognosis in ESCC, and a high expression of BMP1 tended to indicate a lower possibility of distant metastasis. It was reported that the up-regulation of BMP1 may indicate the poor prognosis of gastric cancer (35), osteosarcoma (36), renal clear cell carcinoma (37), and other tumors. Nevertheless, the study of BMP1 in EC has not been reported.

Interleukin1β (IL-1β) is considered an essential regulatory factor that promotes tumor progression, metastasis and immunosuppression (38, 39). In addition, IL could be used as a biomarker for the prognosis of non-small cell lung cancer (40). A team study on the relationship between genetic polymorphisms of IL1A and IL1B and thyroid cancer in the Chinese Han population showed that IL1Ars3783521 was a risk factor for thyroid cancer, and rs3136558 and rs1143623 in the IL1B gene suggested susceptibility to the disease in patients older than 48 years (38). Our pilot study showed that IL-1B was a risk immune-related differential gene for EAC, and its expression was generally higher in females than that in males. It was found in the present study that MAPT expression was associated with poor prognosis in EAC, and its expression in men was higher than that in women. Some studies using the TCGA database found that MAPT gene expression was closely related to survival of patients with low-grade gliomas (41). By analyzing the clinical data of patients with breast cancer, Pan et al. concluded that MAPT-AS1 may be a potential therapeutic target for ER-negative breast cancer related to tumor growth, invasion and drug resistance (42). The prognostic role of MAPT in prostate cancer and childhood blastoma were also proposed in some studies (41).

More importantly, we reported herein two prognostic index models of immune-related genes based on the expression levels and corresponding regression coefficients of specific genes in ESCC and EAC. The risk score of EC patients was calculated to provide reference for predicting the efficacy of immunotherapy in EC patients. According to the score, the patients were divided into a high-risk group and a low-risk group. The results showed that the survival prognosis of the high-risk group of ESCC and EAC was significantly worse than that of the low-risk group, and the higher the risk score, the worse the prognosis. Previous studies explored some markers to predict the efficacy of chemotherapy and radiotherapy in EC patients, but there are few biological indicators to evaluate the efficacy of immunotherapy for the EC subtypes. A study of immune-related genes in EC is similar to ours, but it does not reflect the difference of immune-related genes in different pathological types of EC. In this study, we established a prognostic score model based on nine genes (HSPA6, CACYBP, DKK1, EGF, FGF19, GAST, OSM, ANGPTL3 and NR2F2). We also analyzed the clinical associations of these genes and found that DKK1 was associated with worse T stages of EC. The expression of OSM in patients with tumor stage III and IV was reportedly higher than that in patients with stage I and II (43). Xi et al. developed a prognostic model of EC based on the histological grade, tumor location, baseline PET SUV max and lymph node size and found that it could be used to evaluate whether induction chemotherapy before neoadjuvant radiotherapy and chemotherapy could benefit the patients (44). Wang and his team established a model for predicting the postoperative survival of ESCC by using UBE2C and MGP genes, as well as the clinicopathological factors including the tumor staging and grade (45). A study on the genome of ESCC used data from two clinical centers and found that NFE2L2 may be a tumor suppressor in ESCC. However, its mutation was found to be associated with poor prognosis, and hot spot mutations in the SLC35E2 promoter region also indicated a low survival rate (46).

Besides, We also explored the relationship between our model risk score and immune cell infiltration. The tumor immune microenvironment contains a variety of immune cells including dendritic cell, natural killer cell, macrophage, T and B lymphocyte, all of which can affect the efficacy of ICIs (47). However, we only concluded that the immune differential gene risk score related to prognosis was negatively correlated with B cell infiltration in ESCC. In the study of other immune cells, no correlation was found between the infiltration level and the score of the model. A recent study used tissue microarray of ESCC and EAC to explore changes in immune cell infiltration in the two EC subtypes by the immunohistochemical method. Their results showed that CD45RO+ and CD8+ cells were highly expressed in EC, and the level of invasion in ESCC was higher than that in EAC (48). Guo et al. also created immune-related genes in the prognostic index score in EC. Unlike our results, they found no statistically significant correlation between B cell infiltration and the score, while the positive level of dendritic cell and macrophage neutrophil suggested a higher risk score (43), which is inconsistent with our results. Therefore, we analyzed their research and found that sample differences might cause this inconsistency.

Our work had some potential limitations. First, our research data came from the public databases, which may lack detailed clinical treatment plans and follow-up information in some patients. Second, our study is a retrospective analysis, and it is therefore necessary to verify the prognostic role of these immune-related genes in EC in larger-scale studies. In addition, current work lacked clear evidences that patients with specific scores could or could not benefit from immunotherapy. Finally, we failed to clarify the relationship between immunogenomics, proteomics, and metabonomics and explore the immunobiological mechanism of EC at the molecular level.

## Conclusions

We identified the immune differential genes of different EC subtypes by using the public databases for genome analysis and established a model index of immune differential genes related to prognosis. The results of the present study may provide a new idea for individualized immunotherapy for EC patients.

## Data Availability Statement

The datasets presented in this study can be found in online repositories. The names of the repository/repositories and accession number(s) can be found in the article/supplementary material.

## Author Contributions

YZ and TX conceived and designed the study and assisted in writing the manuscript. ZF and RX performed the data analyses and contributed to the writing of the manuscript. ZC, JX, and YG reviewed the manuscript. All authors contributed to the article and approved the submitted version.

## Funding

This study was supported by grant from the Natural Science Foundation of Zhejiang Province (No. LY21H280012) and the new-shoot Talents Porgram of Zhejiang province (No. 2021).

## Conflict of Interest

The authors declare that the research was conducted in the absence of any commercial or financial relationships that could be construed as a potential conflict of interest.
